# Supplementary material for: Differentiation between myopericarditis and acute myocardial infarction on presentation in the emergency department using the admission C-reactive protein to troponin ratio
Source: PLoS One. 2021 Apr 22;16(4):e0248365. doi: 10.1371/journal.pone.0248365 (PMC8062049; doi:10.1371/journal.pone.0248365)
Supplement: S1 File — (DOCX) [file pone.0248365.s001.docx]

Table 2: The accuracy of CRP/troponin ratio levels measured early on presentation in STEMI with or without NSTEMI patients for distinguishing myopericarditis from myocardial infarction. Asterisks denote Youden values.

Table demonstrates the accuracy of CRP/troponin>30 to diagnose myopericarditis against AMI (STEMI+NSTEMI): For calculation of sensitivity, specificity, PPV and NPV

| **n_Early.CRP/CTN.ratio.501 * n_Group.DX.pericarditis Crosstabulation** | | | | | |  |
| --- | --- | --- | --- | --- | --- | --- |
|  | | | n_Group.DX.pericarditis | | Total |  |
|  |  |  | 1.00 | 3.00=pri |  |  |
| n_Early.CRP.CTN.ratio.501 | 1.00 | Count | 636 | 144 | 780 |  |
|  |  | % within n_Early.CRP.CTN.ratio.501 | 81.5% | 18.5% | 100.0% |  |
|  |  | % within n_Group.DX.pericarditis | 86.9% | 50.0% | 76.5% |  |
|  | 2.00 | Count | 96 | 144 | 240 |  |
|  |  | % within n_Early.CRP.CTN.ratio.501 | 40.0% | 60.0% | 100.0% |  |
|  |  | % within n_Group.DX.pericarditis | 13.1% | 50.0% | 23.5% |  |
| Total | | Count | 732 | 288 | 1020 |  |
|  |  | % within n_Early.CRP.CTN.ratio.501 | 71.8% | 28.2% | 100.0% |  |
|  |  | % within n_Group.DX.pericarditis | 100.0% | 100.0% | 100.0% |  |

| **n_Early.CRP/CTN.ratio.30 * n_Group.DX.pericarditis Crosstabulation** | | | | | |
| --- | --- | --- | --- | --- | --- |
|  | | | n_Group.DX.pericarditis | | Total |
|  |  |  | 1.00 | 3.00=pri |  |
| n_Early.CRP.CTN.ratio.30 | 1.00 | Count | 226 | 32 | 258 |
|  |  | % within n_Early.CRP.CTN.ratio.30 | 87.6% | 12.4% | 100.0% |
|  |  | % within n_Group.DX.pericarditis | 30.9% | 11.1% | 25.3% |
|  | 2.00 | Count | 506 | 256 | 762 |
|  |  | % within n_Early.CRP.CTN.ratio.30 | 66.4% | 33.6% | 100.0% |
|  |  | % within n_Group.DX.pericarditis | 69.1% | 88.9% | 74.7% |
| Total | | Count | 732 | 288 | 1020 |
|  |  | % within n_Early.CRP.CTN.ratio.30 | 71.8% | 28.2% | 100.0% |
|  |  | % within n_Group.DX.pericarditis | 100.0% | 100.0% | 100.0% |

| Table demonstrates the accuracy of CRP/troponin>60 to diagnose myopericarditis against AMI (STEMI+NSTEMI)  **n_Early.CRP/CTN.ratio.60 * n_Group.DX.pericarditis Crosstabulation** | | | | | |
| --- | --- | --- | --- | --- | --- |
|  | | | n_Group.DX.pericarditis | | Total |
|  |  |  | 1.00 | 3.00=pri |  |
| n_Early.CRP.CTN.ratio.60 | 1.00 | Count | 329 | 44 | 373 |
|  |  | % within n_Early.CRP.CTN.ratio.60 | 88.2% | 11.8% | 100.0% |
|  |  | % within n_Group.DX.pericarditis | 44.9% | 15.3% | 36.6% |
|  | 2.00 | Count | 403 | 244 | 647 |
|  |  | % within n_Early.CRP.CTN.ratio.60 | 62.3% | 37.7% | 100.0% |
|  |  | % within n_Group.DX.pericarditis | 55.1% | 84.7% | 63.4% |
| Total | | Count | 732 | 288 | 1020 |
|  |  | % within n_Early.CRP.CTN.ratio.60 | 71.8% | 28.2% | 100.0% |
|  |  | % within n_Group.DX.pericarditis | 100.0% | 100.0% | 100.0% |

Table demonstrates the accuracy of CRP/troponin>100 to diagnose myopericarditis against AMI (STEMI+NSTEMI)

| **n_Early.CRP/CTN.ratio.100 * n_Group.DX.pericarditis Crosstabulation** | | | | | |
| --- | --- | --- | --- | --- | --- |
|  | | | n_Group.DX.pericarditis | | Total |
|  |  |  | 1.00 | 3.00=pri |  |
| n_Early.CRP.CTN.ratio.100 | 1.00 | Count | 409 | 71 | 480 |
|  |  | % within n_Early.CRP.CTN.ratio.100 | 85.2% | 14.8% | 100.0% |
|  |  | % within n_Group.DX.pericarditis | 56.0% | 24.7% | 47.1% |
|  | 2.00 | Count | 322 | 217 | 539 |
|  |  | % within n_Early.CRP.CTN.ratio.100 | 59.7% | 40.3% | 100.0% |
|  |  | % within n_Group.DX.pericarditis | 44.0% | 75.3% | 52.9% |
| Total | | Count | 731 | 288 | 1019 |
|  |  | % within n_Early.CRP.CTN.ratio.100 | 71.7% | 28.3% | 100.0% |
|  |  | % within n_Group.DX.pericarditis | 100.0% | 100.0% | 100.0% |

Table demonstrates the accuracy of CRP/troponin>150 to diagnose myopericarditis against AMI (STEMI+NSTEMI)

| **n_Early.CRP/CTN.ratio.150 * n_Group.DX.pericarditis Crosstabulation** | | | | | |
| --- | --- | --- | --- | --- | --- |
|  | | | n_Group.DX.pericarditis | | Total |
|  |  |  | 1.00 | 3.00=pri |  |
| n_Early.CRP.CTN.ratio.150 | 1.00 | Count | 466 | 89 | 555 |
|  |  | % within n_Early.CRP.CTN.ratio.150 | 84.0% | 16.0% | 100.0% |
|  |  | % within n_Group.DX.pericarditis | 63.7% | 30.9% | 54.4% |
|  | 2.00 | Count | 266 | 199 | 465 |
|  |  | % within n_Early.CRP.CTN.ratio.150 | 57.2% | 42.8% | 100.0% |
|  |  | % within n_Group.DX.pericarditis | 36.3% | 69.1% | 45.6% |
| Total | | Count | 732 | 288 | 1020 |
|  |  | % within n_Early.CRP.CTN.ratio.150 | 71.8% | 28.2% | 100.0% |
|  |  | % within n_Group.DX.pericarditis | 100.0% | 100.0% | 100.0% |

Table demonstrates the accuracy of CRP/troponin>300 to diagnose myopericarditis against AMI (STEMI+NSTEMI)

| **n_Early.CRP/CTN.ratio.300 * n_Group.DX.pericarditis Crosstabulation** | | | | | |
| --- | --- | --- | --- | --- | --- |
|  | | | n_Group.DX.pericarditis | | Total |
|  |  |  | 1.00 | 3.00=pri |  |
| n_Early.CRP.CTN.ratio.300 | 1.00 | Count | 561 | 126 | 687 |
|  |  | % within n_Early.CRP.CTN.ratio.300 | 81.7% | 18.3% | 100.0% |
|  |  | % within n_Group.DX.pericarditis | 76.6% | 43.8% | 67.4% |
|  | 2.00 | Count | 171 | 162 | 333 |
|  |  | % within n_Early.CRP.CTN.ratio.300 | 51.4% | 48.6% | 100.0% |
|  |  | % within n_Group.DX.pericarditis | 23.4% | 56.3% | 32.6% |
| Total | | Count | 732 | 288 | 1020 |
|  |  | % within n_Early.CRP.CTN.ratio.300 | 71.8% | 28.2% | 100.0% |
|  |  | % within n_Group.DX.pericarditis | 100.0% | 100.0% | 100.0% |

| Table demonstrates the accuracy of CRP/troponin>1000 to diagnose myopericarditis against AMI (STEMI+NSTEMI)  **n_Early.CRP/CTN.ratio.1000 * n_Group.DX.pericarditis Crosstabulation** | | | | | |
| --- | --- | --- | --- | --- | --- |
|  | | | n_Group.DX.pericarditis | | Total |
|  |  |  | 1.00 | 3.00 |  |
| n_Early.CRP.CTN.ratio.1000 | 1.00 | Count | 688 | 173 | 861 |
|  |  | % within n_Early.CRP.CTN.ratio.1000 | 79.9% | 20.1% | 100.0% |
|  |  | % within n_Group.DX.pericarditis | 94.0% | 60.1% | 84.4% |
|  | 2.00 | Count | 44 | 115 | 159 |
|  |  | % within n_Early.CRP.CTN.ratio.1000 | 27.7% | 72.3% | 100.0% |
|  |  | % within n_Group.DX.pericarditis | 6.0% | 39.9% | 15.6% |
| Total | | Count | 732 | 288 | 1020 |
|  |  | % within n_Early.CRP.CTN.ratio.1000 | 71.8% | 28.2% | 100.0% |
|  |  | % within n_Group.DX.pericarditis | 100.0% | 100.0% | 100.0% |

CRP/Troponin in myopericarditis vs. NSTEMI

| **n_Early.CRP.CTN.ratio414 * group.DX Crosstabulation** | | | | | |
| --- | --- | --- | --- | --- | --- |
| n_Early.CRP.CTN.ratio414 | | | group.DX | | Total |
|  |  |  | NON STEIMI | PERICARDITIS |  |
|  | 1.00 | Count | 226 | 136 | 362 |
|  |  | % within n_Early.CRP.CTN.ratio414 | 62.4% | 37.6% | 100.0% |
|  |  | % within group.DX | 87.6% | 47.2% | 66.3% |
|  | 2.00 | Count | 32 | 152 | 184 |
|  |  | % within n_Early.CRP.CTN.ratio414 | 17.4% | 82.6% | 100.0% |
|  |  | % within group.DX | 12.4% | 52.8% | 33.7% |
| Total | | Count | 258 | 288 | 546 |
|  |  | % within n_Early.CRP.CTN.ratio414 | 47.3% | 52.7% | 100.0% |
|  |  | % within group.DX | 100.0% | 100.0% | 100.0% |

| **n_Early.CRP/CTN.ratio.30 * group.DX Crosstabulation** | | | | | |
| --- | --- | --- | --- | --- | --- |
| n_Early.CRP.CTN.ratio.30 | | | group.DX | | Total |
|  |  |  | NON STEIMI | PERICARDITIS |  |
|  | 1.00 | Count | 81 | 32 | 113 |
|  |  | % within n_Early.CRP.CTN.ratio.30 | 71.7% | 28.3% | 100.0% |
|  |  | % within group.DX | 31.4% | 11.1% | 20.7% |
|  | 2.00 | Count | 177 | 256 | 433 |
|  |  | % within n_Early.CRP.CTN.ratio.30 | 40.9% | 59.1% | 100.0% |
|  |  | % within group.DX | 68.6% | 88.9% | 79.3% |
| Total | | Count | 258 | 288 | 546 |
|  |  | % within n_Early.CRP.CTN.ratio.30 | 47.3% | 52.7% | 100.0% |
|  |  | % within group.DX | 100.0% | 100.0% | 100.0% |

| **n_Early.CRP/CTN.ratio.60 * group.DX Crosstabulation** | | | | | |
| --- | --- | --- | --- | --- | --- |
| n_Early.CRP.CTN.ratio.60 | | | group.DX | | Total |
|  |  |  | NON STEIMI | PERICARDITIS |  |
|  | 1.00 | Count | 121 | 44 | 165 |
|  |  | % within n_Early.CRP.CTN.ratio.60 | 73.3% | 26.7% | 100.0% |
|  |  | % within group.DX | 46.9% | 15.3% | 30.2% |
|  | 2.00 | Count | 137 | 244 | 381 |
|  |  | % within n_Early.CRP.CTN.ratio.60 | 36.0% | 64.0% | 100.0% |
|  |  | % within group.DX | 53.1% | 84.7% | 69.8% |
| Total | | Count | 258 | 288 | 546 |
|  |  | % within n_Early.CRP.CTN.ratio.60 | 47.3% | 52.7% | 100.0% |
|  |  | % within group.DX | 100.0% | 100.0% | 100.0% |

| **n_Early.CRP/CTN.ratio.100 * group.DX Crosstabulation** | | | | | |
| --- | --- | --- | --- | --- | --- |
| n_Early.CRP.CTN.ratio.100 | | | group.DX | | Total |
|  |  |  | NON STEIMI | PERICARDITIS |  |
|  | 1.00 | Count | 155 | 71 | 226 |
|  |  | % within n_Early.CRP.CTN.ratio.100 | 68.6% | 31.4% | 100.0% |
|  |  | % within group.DX | 60.1% | 24.7% | 41.4% |
|  | 2.00 | Count | 103 | 217 | 320 |
|  |  | % within n_Early.CRP.CTN.ratio.100 | 32.2% | 67.8% | 100.0% |
|  |  | % within group.DX | 39.9% | 75.3% | 58.6% |
| Total | | Count | 258 | 288 | 546 |
|  |  | % within n_Early.CRP.CTN.ratio.100 | 47.3% | 52.7% | 100.0% |
|  |  | % within group.DX | 100.0% | 100.0% | 100.0% |

| **n_Early.CRP/CTN.ratio.150 * group.DX Crosstabulation** | | | | | |
| --- | --- | --- | --- | --- | --- |
| n_Early.CRP.CTN.ratio.150 | | | group.DX | | Total |
|  |  |  | NON STEIMI | PERICARDITIS |  |
|  | 1.00 | Count | 175 | 89 | 264 |
|  |  | % within n_Early.CRP.CTN.ratio.150 | 66.3% | 33.7% | 100.0% |
|  |  | % within group.DX | 67.8% | 30.9% | 48.4% |
|  | 2.00 | Count | 83 | 199 | 282 |
|  |  | % within n_Early.CRP.CTN.ratio.150 | 29.4% | 70.6% | 100.0% |
|  |  | % within group.DX | 32.2% | 69.1% | 51.6% |
| Total | | Count | 258 | 288 | 546 |
|  |  | % within n_Early.CRP.CTN.ratio.150 | 47.3% | 52.7% | 100.0% |
|  |  | % within group.DX | 100.0% | 100.0% | 100.0% |

| **n_Early.CRP/CTN.ratio.300 * group.DX Crosstabulation** | | | | | |
| --- | --- | --- | --- | --- | --- |
| n_Early.CRP.CTN.ratio.300 | | | group.DX | | Total |
|  |  |  | NON STEIMI | PERICARDITIS |  |
|  | 1.00 | Count | 209 | 126 | 335 |
|  |  | % within n_Early.CRP.CTN.ratio.300 | 62.4% | 37.6% | 100.0% |
|  |  | % within group.DX | 81.0% | 43.8% | 61.4% |
|  | 2.00 | Count | 49 | 162 | 211 |
|  |  | % within n_Early.CRP.CTN.ratio.300 | 23.2% | 76.8% | 100.0% |
|  |  | % within group.DX | 19.0% | 56.3% | 38.6% |
| Total | | Count | 258 | 288 | 546 |
|  |  | % within n_Early.CRP.CTN.ratio.300 | 47.3% | 52.7% | 100.0% |
|  |  | % within group.DX | 100.0% | 100.0% | 100.0% |

CRP/Troponin in myopericarditis vs. STEMI

| **n_Early.CRP.CTN.ratio.513 * group.DX Crosstabulation** | | | | | |
| --- | --- | --- | --- | --- | --- |
| n_Early.CRP.CTN.ratio.513 | | | group.DX | | Total |
|  |  |  | STEMI | PERICARDITIS |  |
|  | 1.00 | Count | 406 | 145 | 551 |
|  |  | % within n_Early.CRP.CTN.ratio.513 | 73.7% | 26.3% | 100.0% |
|  |  | % within group.DX | 85.7% | 50.3% | 72.3% |
|  | 2.00 | Count | 68 | 143 | 211 |
|  |  | % within n_Early.CRP.CTN.ratio.513 | 32.2% | 67.8% | 100.0% |
|  |  | % within group.DX | 14.3% | 49.7% | 27.7% |
| Total | | Count | 474 | 288 | 762 |
|  |  | % within n_Early.CRP.CTN.ratio.513 | 62.2% | 37.8% | 100.0% |
|  |  | % within group.DX | 100.0% | 100.0% | 100.0% |

| **n_Early.CRP/CTN.ratio.30 * group.DX Crosstabulation** | | | | | |
| --- | --- | --- | --- | --- | --- |
| n_Early.CRP.CTN.ratio.30 | | | group.DX | | Total |
|  |  |  | STEMI | PERICARDITIS |  |
|  | 1.00 | Count | 145 | 32 | 177 |
|  |  | % within n_Early.CRP.CTN.ratio.30 | 81.9% | 18.1% | 100.0% |
|  |  | % within group.DX | 30.6% | 11.1% | 23.2% |
|  | 2.00 | Count | 329 | 256 | 585 |
|  |  | % within n_Early.CRP.CTN.ratio.30 | 56.2% | 43.8% | 100.0% |
|  |  | % within group.DX | 69.4% | 88.9% | 76.8% |
| Total | | Count | 474 | 288 | 762 |
|  |  | % within n_Early.CRP.CTN.ratio.30 | 62.2% | 37.8% | 100.0% |
|  |  | % within group.DX | 100.0% | 100.0% | 100.0% |

| **n_Early.CRP/CTN.ratio.60 * group.DX Crosstabulation** | | | | | |
| --- | --- | --- | --- | --- | --- |
| n_Early.CRP.CTN.ratio.60 | | | group.DX | | Total |
|  |  |  | STEMI | PERICARDITIS |  |
|  | 1.00 | Count | 208 | 44 | 252 |
|  |  | % within n_Early.CRP.CTN.ratio.60 | 82.5% | 17.5% | 100.0% |
|  |  | % within group.DX | 43.9% | 15.3% | 33.1% |
|  | 2.00 | Count | 266 | 244 | 510 |
|  |  | % within n_Early.CRP.CTN.ratio.60 | 52.2% | 47.8% | 100.0% |
|  |  | % within group.DX | 56.1% | 84.7% | 66.9% |
| Total | | Count | 474 | 288 | 762 |
|  |  | % within n_Early.CRP.CTN.ratio.60 | 62.2% | 37.8% | 100.0% |
|  |  | % within group.DX | 100.0% | 100.0% | 100.0% |

| **n_Early.CRP/CTN.ratio.100 * group.DX Crosstabulation** | | | | | |
| --- | --- | --- | --- | --- | --- |
| n_Early.CRP.CTN.ratio.100 | | | group.DX | | Total |
|  |  |  | STEMI | PERICARDITIS |  |
|  | 1.00 | Count | 254 | 71 | 325 |
|  |  | % within n_Early.CRP.CTN.ratio.100 | 78.2% | 21.8% | 100.0% |
|  |  | % within group.DX | 53.7% | 24.7% | 42.7% |
|  | 2.00 | Count | 219 | 217 | 436 |
|  |  | % within n_Early.CRP.CTN.ratio.100 | 50.2% | 49.8% | 100.0% |
|  |  | % within group.DX | 46.3% | 75.3% | 57.3% |
| Total | | Count | 473 | 288 | 761 |
|  |  | % within n_Early.CRP.CTN.ratio.100 | 62.2% | 37.8% | 100.0% |
|  |  | % within group.DX | 100.0% | 100.0% | 100.0% |

| **n_Early.CRP/CTN.ratio.150 * group.DX Crosstabulation** | | | | | |
| --- | --- | --- | --- | --- | --- |
| n_Early.CRP.CTN.ratio.150 | | | group.DX | | Total |
|  |  |  | STEMI | PERICARDITIS |  |
|  | 1.00 | Count | 291 | 89 | 380 |
|  |  | % within n_Early.CRP.CTN.ratio.150 | 76.6% | 23.4% | 100.0% |
|  |  | % within group.DX | 61.4% | 30.9% | 49.9% |
|  | 2.00 | Count | 183 | 199 | 382 |
|  |  | % within n_Early.CRP.CTN.ratio.150 | 47.9% | 52.1% | 100.0% |
|  |  | % within group.DX | 38.6% | 69.1% | 50.1% |
| Total | | Count | 474 | 288 | 762 |
|  |  | % within n_Early.CRP.CTN.ratio.150 | 62.2% | 37.8% | 100.0% |
|  |  | % within group.DX | 100.0% | 100.0% | 100.0% |

| **n_Early.CRP/CTN.ratio.300 * group.DX Crosstabulation** | | | | | |
| --- | --- | --- | --- | --- | --- |
| n_Early.CRP.CTN.ratio.300 | | | group.DX | | Total |
|  |  |  | STEMI | PERICARDITIS |  |
|  | 1.00 | Count | 352 | 126 | 478 |
|  |  | % within n_Early.CRP.CTN.ratio.300 | 73.6% | 26.4% | 100.0% |
|  |  | % within group.DX | 74.3% | 43.8% | 62.7% |
|  | 2.00 | Count | 122 | 162 | 284 |
|  |  | % within n_Early.CRP.CTN.ratio.300 | 43.0% | 57.0% | 100.0% |
|  |  | % within group.DX | 25.7% | 56.3% | 37.3% |
| Total | | Count | 474 | 288 | 762 |
|  |  | % within n_Early.CRP.CTN.ratio.300 | 62.2% | 37.8% | 100.0% |
|  |  | % within group.DX | 100.0% | 100.0% | 100.0% |

| **n_Early.CRP/CTN.ratio.1000 * group.DX Crosstabulation** | | | | | |
| --- | --- | --- | --- | --- | --- |
| n_Early.CRP.CTN.ratio.1000 | | | group.DX | | Total |
|  |  |  | STEMI | PERICARDITIS |  |
|  | 1.00 | Count | 437 | 173 | 610 |
|  |  | % within n_Early.CRP.CTN.ratio.1000 | 71.6% | 28.4% | 100.0% |
|  |  | % within group.DX | 92.2% | 60.1% | 80.1% |
|  | 2.00 | Count | 37 | 115 | 152 |
|  |  | % within n_Early.CRP.CTN.ratio.1000 | 24.3% | 75.7% | 100.0% |
|  |  | % within group.DX | 7.8% | 39.9% | 19.9% |
| Total | | Count | 474 | 288 | 762 |
|  |  | % within n_Early.CRP.CTN.ratio.1000 | 62.2% | 37.8% | 100.0% |
|  |  | % within group.DX | 100.0% | 100.0% | 100.0% |

| **n_Early.CRP/CTN.ratio.1000 * group.DX Crosstabulation** | | | | | |
| --- | --- | --- | --- | --- | --- |
| n_Early.CRP.CTN.ratio.1000 | | | group.DX | | Total |
|  |  |  | NON STEIMI | PERICARDITIS |  |
|  | 1.00 | Count | 251 | 173 | 424 |
|  |  | % within n_Early.CRP.CTN.ratio.1000 | 59.2% | 40.8% | 100.0% |
|  |  | % within group.DX | 97.3% | 60.1% | 77.7% |
|  | 2.00 | Count | 7 | 115 | 122 |
|  |  | % within n_Early.CRP.CTN.ratio.1000 | 5.7% | 94.3% | 100.0% |
|  |  | % within group.DX | 2.7% | 39.9% | 22.3% |
| Total | | Count | 258 | 288 | 546 |
|  |  | % within n_Early.CRP.CTN.ratio.1000 | 47.3% | 52.7% | 100.0% |
|  |  | % within group.DX | 100.0% | 100.0% | 100.0% |


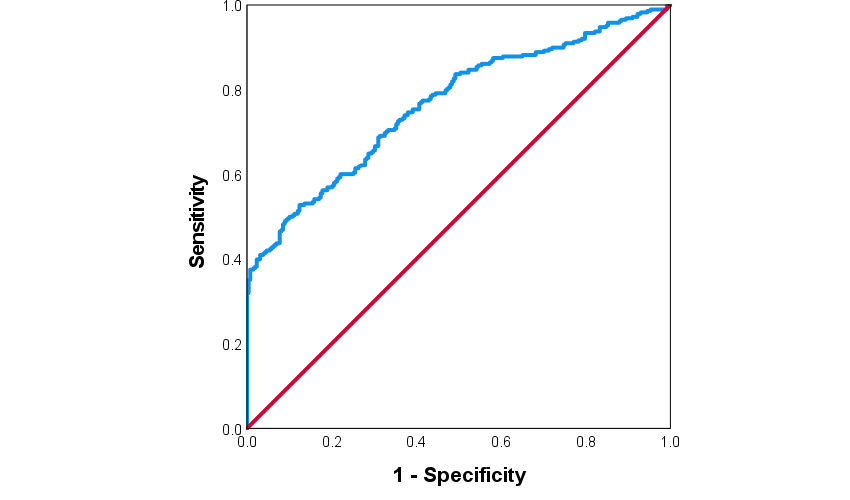


| **Area Under the Curve** | | | | |
| --- | --- | --- | --- | --- |
| Test Result Variable(s): Early.CRP_cTn.ratio – pericarditisvs. Non stemi | | | | |
| Area | Std. Error^a^ | p-value | Asymptotic 95% Confidence Interval | |
|  |  |  | Lower Bound | Upper Bound |
| .765 | .020 | .000 | .726 | .804 |
|  | | | | |
|  | | | | |

– לפי רבעונים של המנהThe multivariable model to predict myopericarditis against NSTEMI or STEMI by quartiles of CRP/troponin

| **Categorical Variables Codings** | | | | | |
| --- | --- | --- | --- | --- | --- |
|  | | Frequency | Parameter coding | | |
|  |  |  | (1) | (2) | (3) |
| n_ratio.IQR | Lowest thru 29.41 | 255 | .000 | .000 | .000 |
|  | 29.4118 thru 116.5669 | 255 | 1.000 | .000 | .000 |
|  | 116.56691 thru 455 | 255 | .000 | 1.000 | .000 |
|  | (455.1 thru Highest | 253 | .000 | .000 | 1.000 |

| **Variables in the Equation** | | | | | | | |
| --- | --- | --- | --- | --- | --- | --- | --- |
|  | | B | Sig. | Exp(B) | 95% C.I.for EXP(B) | |  |
|  |  |  |  |  | Lower | Upper |  |
| Step 1^a^ | Age | -.070 | .000 | .933 | .922 | .944 |  |
|  | Creat | .206 | .043 | 1.229 | 1.006 | 1.502 |  |
|  | n_ratio.IQR |  | .000 |  |  |  |  |
|  | n_ratio.IQR(1) | .585 | .034 | 1.795 | 1.045 | 3.085 |  |
|  | n_ratio.IQR(2) | .842 | .001 | 2.321 | 1.385 | 3.890 |  |
|  | n_ratio.IQR(3) | 2.333 | .000 | 10.309 | 6.259 | 16.981 |  |
|  | Constant | 1.544 | .000 | 4.685 |  |  |  |

Increasing age decreases probability of myopericarditis. Each 1-year increase decreases probability by 0.07. Odds ratio=0.933, p<0.0001 and 95%CI=0.922-0.944

Each unit increase in creatinine unit increases probability of myopericarditis. Odds ratio=1.229, p=0.043 and 95%CI=1.006-1.502.

Reference: First quartile as the

Odds ratio-1.795, p=0.034 and 95%CI=1.045-3.085 Second quartile:

Third quartile odds ratio=2.321, p=0.001 and 95%CI=1.385-3.890

Fourth quartile odds ratio=10.309, p<0.0001 and 95%CI=6.259-16.981

Age effect by quartiles

| **Categorical Variables Codings** | | | | | |
| --- | --- | --- | --- | --- | --- |
|  | | Frequency | Parameter coding | | |
|  |  |  | (1) | (2) | (3) |
| n_age.IQR | age<50 | 290 | .000 | .000 | .000 |
|  | age 50-59 | 246 | 1.000 | .000 | .000 |
|  | 59-69 | 233 | .000 | 1.000 | .000 |
|  | 69+ | 249 | .000 | .000 | 1.000 |
|  |  |  |  |  |  |
| n_ratio.IQR | Lowest thru 29.41 | 255 | .000 | .000 | .000 |
|  | 29.4118 thru 116.5669 | 255 | 1.000 | .000 | .000 |
|  | 116.56691 thru 455 | 255 | .000 | 1.000 | .000 |
|  | (455.1 thru Highest | 253 | .000 | .000 | 1.000 |

| **Variables in the Equation** | | | | | | | |
| --- | --- | --- | --- | --- | --- | --- | --- |
|  | | B | p-value | Odds ratio | 95% C.I.for EXP(B) | |  |
|  |  |  |  |  | Lower | Upper |  |
| Step 1^a^ | n_ratio.IQR |  | .000 |  |  |  |  |
|  | n_ratio.IQR(1) | .431 | .108 | 1.539 | .910 | 2.604 |  |
|  | n_ratio.IQR(2) | .715 | .005 | 2.045 | 1.237 | 3.381 |  |
|  | n_ratio.IQR(3) | 2.311 | .000 | 10.085 | 6.188 | 16.436 |  |
|  | n_age.IQR |  | .000 |  |  |  |  |
|  | n_age.IQR(1) | -2.139 | .000 | .118 | .075 | .186 |  |
|  | n_age.IQR(2) | -1.921 | .000 | .146 | .093 | .231 |  |
|  | n_age.IQR(3) | -1.922 | .000 | .146 | .093 | .231 |  |
|  | Creat | .073 | .516 | 1.076 | .863 | 1.341 |  |
|  | Constant | -.725 | .004 | .484 |  |  |  |

Logistic regression analysis was performed with age quartiles (<50, 50-59, 59-60, >69 years) and quartiles of CRP/troponin ratio (<29, 29-117, 117-455, >455) and creatinine level as multivariables for prediction of myopericarditis. The second to third age quartile compared with the first quartile were negatively associated with myopericarditis (all p<0.0001). The third and fourth quartiles compared to the first quartile were statistically significantly associated with myopericarditis (p<0.005, p<0.0001). There was no correlation between creatinine concentration and myopericarditis (p=0.52).

USE ALL.

COMPUTE filter_$=(group.DX < 4).

RECODE Early.CRP_cTn.ratio:

(Lowest thru 8.641=1)

(8.64101 thru 22.8289=2)

(22.82891 thru 41.7697=3)

(41.76971 thru 69.7040=4)

(69.70401 thru 115.8483=5)

(115.84831 thru 199.1837=6)

(199.18371 thru 346.1538=7)

(346.15381 thru 657.8365=8)

(657.83651 thru 2309.1429=9)

(2309.14291 thru Highest=10)

INTO n_Early.CRP_cTn.ratio.percentiles.
